# COL11A1 as an novel biomarker for breast cancer with machine learning and immunohistochemistry validation

**DOI:** 10.3389/fimmu.2022.937125

**Published:** 2022-10-31

**Authors:** Wenjie Shi, Zhilin Chen, Hui Liu, Chen Miao, Ruifa Feng, Guilin Wang, Guoping Chen, Zhitong Chen, Pingming Fan, Weiyi Pang, Chen Li

**Affiliations:** ^1^ University Hospital for Gynecology, Pius-Hospital, University Medicine Oldenburg, Oldenburg, Germany; ^2^ University Clinic for General, Visceral, Vascular and Transplantation Surgery, Faculty of Medicine, Otto-von-Guericke-University, Magdeburg, Germany; ^3^ Department of Breast Surgery, Hainan Medical University, Haikou, China; ^4^ Guangxi Key Laboratory of Environmental Exposomics and Entire Lifecycle Heath, Guilin Medical University, Guilin, China; ^5^ Department of Pathology, The First Affiliated Hospital of Nanjing Medical University, Nanjing, China; ^6^ Breast Center of The Second Affiliated Hospital of Guilin Medical University, Guilin, China; ^7^ Department of Biology, Chemistry, Pharmacy, Free University of Berlin, Berlin, Germany

**Keywords:** machine learning, COL11A1, breast cancer, tumor microenvironment, prognosis

## Abstract

Machine learning (ML) algorithms were used to identify a novel biological target for breast cancer and explored its relationship with the tumor microenvironment (TME) and patient prognosis. The edgR package identified hub genes associated with overall survival (OS) and prognosis, which were validated using public datasets. Of 149 up-regulated genes identified in tumor tissues, three ML algorithms identified COL11A1 as a hub gene. COL11A1was highly expressed in breast cancer samples and associated with a poor prognosis, and positively correlated with a stromal score (r=0.49, p<0.001) and the ESTIMATE score (r=0.29, p<0.001) in the TME. Furthermore, COL11A1 negatively correlated with B cells, CD4 and CD8 cells, but positively associated with cancer-associated fibroblasts. Forty-three related immune-regulation genes associated with COL11A1 were identified, and a five-gene immune regulation signature was built. Compared with clinical factors, this gene signature was an independent risk factor for prognosis (HR=2.591, 95%CI 1.831–3.668, p=7.7e-08). A nomogram combining the gene signature with clinical variables, showed better predictive performance (C-index=0.776). The model correction prediction curve showed little bias from the ideal curve. COL11A1 is a potential therapeutic target in breast cancer and may be involved in the tumor immune infiltration; its high expression is strongly associated with poor prognosis.

## Background

Breast cancer is one of the most commonly diagnosed malignant tumors in the world. As the most frequent malignant tumor in women, more than 2.1 million women have been diagnosed with breast cancer in 2018, and approximately 500,000 women have died from this disease (1, 2). Although advances in early detection and effective systemic treatment have decreased breast cancer mortality rates in North America and the European Union, breast cancer remains the most common cause of cancer death in less developed countries, second only to lung cancer, and almost all patients in the advanced stage have a poor prognosis (3). Therefore, new therapeutic approaches and goals need to be developed to reduce disease recurrence and death.

With the advances in machine learning, we have achieved great success for disease diagnosis, risk stratification, and the establishment of prognostic models (4), such as using medical imaging and artificial intelligence for the identification of lesions (5–7), the discovery of new biomarkers through data mining, drug discovery, and risk model construction (8, 9). Traditionally, machine learning approaches are divided into supervised learning, unsupervised learning, and reinforcement learning categories. We can predict and classify huge data using machine learning algorithms based on known training data. As reported by Rahman (10), Linear Regression (LR), Support Vector Machine (SVM), Multi-Layer Perceptron (MLP), and Vector Auto-Regression have been the most widely used algorithms for tackling the Coronavirus pandemic (COVID-19). Thus, our aim was to identify potential prognosis-related biomarkers in breast cancer by computational approaches to assist clinical decision-making.

In recent years, immunotherapy has emerged as a novel option for a variety of solid tumors (11). Unlike other solid cancers, breast cancer is insensitive to immunotherapy. While the recognition of the importance of the tumor microenvironment (TME) in breast cancer progression, response to treatment, and resistance, the assessment of its immune infiltration and stromal cell infiltration has opened the opportunity for breast cancer immunotherapy. Retrospective studies have shown that patients with breast cancer with higher levels of stromal-infiltrating immune cells generally have longer progression-free survival (PFS) and overall survival (OS) (12, 13), and the results of immune checkpoint inhibitor (ICI) therapies for TNbreast cancer are encouraging (14, 15). Studies are ongoing to unravel the immunoediting function of the host immune system in breast cancer to identify patients who will benefit from therapy (16, 17).

Collagen type XI alpha 1 (COL11A1) is a type XI collagen, which belongs to the collagen family. Although it is mainly involved in the biological process of bone development (18), high levels of COL11A1 are associated with tumor metastasis, treatment resistance, and poor clinical outcome in several solid tumors types such as breast, pancreas, and colorectal cancers (19, 20). Gu et al. (21) showed that COL11A1 was highly expressed in breast cancer tissues, and COL11A1 variant E was also significantly correlated with lymph nodes involvement and metastasis in breast cancers (20). As an important component of the structure of the extracellular matrix (ECM), COL11A1 was identified as a correlated predictor of dangerous immune infiltrates in pancreatic adenocarcinoma (22). However, the role of COL11A1 in the TME of breast cancers remains unclear.

## Materials and methods

### Data sourcing and pre-processing

In this study a total of six breast cancer datasets were included. Clinical and expression profile data of patients originating from The Cancer Genome Atlas Program (TCGA) dataset were downloaded using the TCGA Biolink package (23). GSE42568, GSE109169, GSE138536, GSE173839, and GSE103668 were derived from the GEO database. GSE42568, includes 104 breast cancer and 17 normal breast biopsies, GSE109169, includes 25 paired breast samples. GSE138536 is a single-cell sequencing data containing 8 breast cancer samples. GSE173839 and GSE103668, includes follow-up information of breast cancer patients receiving immunotherapy. Cell line expression and protein level expression data were obtained from the Cancer Cell Line Encyclopedia (CCLE) and the Clinical Proteomic Tumor Analysis Consortium (CPTAC) databases. Immune infiltration scores were evaluated using the R package ESTIMATE.

### Differential gene analysis of samples

We performed a differential gene analysis of the breast cancer expression profile data comparing TCGA datasets of tumor and normal tissues using the edgeR package, and the threshold criteria were |LogFC| >4, and the adjust p-value less than 0.01.

### Machine learning identifies feature genes

We first defined patients with an OS shorter than 3 years as the short-term survival group, while those with a survival time greater than 3 years were defined as the long-term survival group. We used the random survival forest to identify short-term related feature genes (24). Machine learning algorithm lasso regression, and Support Vector Machine (SVM) were used to select feature genes (25). Variables of greater importance than 0.3 in random forests were defined as significant. The lambda with the smallest value was defined as significant for lasso regression. For the SVM algorithm, the top 10 feature support vectors were defined as the important variables. The intersection genes of the three machine learning algorithms were defined as the core genes.

### Validated expression of hub gene

Samples from TCGA and GTEX databases were used to validate the expression of the hub gene at the transcriptome level. GSE42568 and GSE109169 were also used to validate the expression differences of COL11A1 in tumor and normal tissues. Data from the CCLE database were used to validate gene expression differences between different cell lines. The protein-level expression differences of COL11A1 were performed through the CPTAC database.

### Analysis of the prognosis value of the hub gene

To further validate the prognostic value of the core gene, we evaluated the association of the expression of the hub gene on OS, DSS and PFS, respectively, using the R survival package. The cut-off values of the patient subgroups were performed using the R package survminer and component differences were obtained by the log-rank test.

### Role of the hub gene in the TME

The level of immune infiltration was evaluated by the ESTIMATE package, which calculated a stromal score and estimated score in each sample, according to gene expression. Additionally, the IOBR package calculated B cell, cancer-associated fibroblasts (CAF), CD4 T cell, CD8 T cell, endothelial cell, macrophage, natural killer cell, and other cell infiltration scores. The Spearman’s test was used to calculate detailed correlations between core genes and B-cell, CD4 and CD8 immune cell markers. Evaluation of prognosis was associated with the level immune infiltration was performed through the TIMER2 website (26).

### Hub gene and relationship with cancer-associated fibroblasts

CAFs play an important role in tumor recurrence and resistance to therapy, as the main component of the tumor stroma. Therefore, we further evaluated the correlation of the hub gene with tumor-associated fibroblasts. We first validated the differential expression of this gene in different cell clusters in GSE138536, a single-cell data set. We then calculated the correlation between the hub gene and the classical fibroblast-associated markers, and finally, we evaluated the association between the level of infiltration and the clinical prognosis.

### Hub gene and relationship with immunotherapy

Immunotherapy offers a new pathway for patients, but not all patients can benefit from this option, and screening of the potentially benefitting population is necessary. Considering that immune checkpoints play an important role in tumor immunotherapy, we first examined the correlation between core genes and immune checkpoints using the Pearson’s test. Then two breast cancer immunotherapy datasets, GSE173839 and GSE103668, containing follow-up information, were interrogated to verify the differential expression of the hub gene between the immune response and immune tolerance groups. Additionally, we analyzed the correlation of this gene with 21 genes related to m6A methylation.

### COL11A1-related immune regulation genes

We extracted the expression data of COL11A1 and 150 immune regulation genes, including chemokines (27), receptors (18), MHC (21), immunoinhibitory genes (24), immunosuppressive genes (46). The Pearson’s correlation between COL11A1 and immune regulation genes was further calculated.

### Prognosis signature construction and validation for OS

Immune regulation genes associated with COL11A1 were put into univariate and multivariate Cox regressions with OS. Univariate significance genes were included in multivariate Cox regression. Then a prognostic signature model was constructed based on the multivariate Cox regression coefficients. An area under the ROC curve (AUC) was used to test the predictive efficiency of the model.

### Nomogram construction and validation

To assess whether the signature had an independent prognostic value compared to other clinical variables, we performed univariate and multivariate cox regression analyses and visualized the regression results to construct a Nomogram model, and the C-index and calibration curve were used to evaluate the predictive efficacy and stability of the model, respectively.

### Immunohistochemistry of COL11A1 between normal and tumor breast tissues

The tissues were washed with PBS and then incubated with 3% H2O2 for 10 min. The antibody including against COL11A1 (1:100, 21841-1-AP, proteintech, CA) were incubated at room temperature for 2 h. After incubation with polymer enhancer for 20 min, the tissue was incubated with polymer enhancer and enzyme-labeled rabbit polymers. Slides were washed with PBS and fresh diaminobenzidine, counterstained with hematoxylin, antigen retrieval performed using 0.1% HCl, dehydrated with ethanol, cleaned with xylene, and fixed with neutral balata. The results were observed and photographed using a fluorescence microscope and visualized under a light microscope at 100× and 200x magnification by a blinded observer. Controls without primary antibodies showed no immunolabeling. Light to dark brown staining indicated a positive result.

## Results

**Figure 1:**
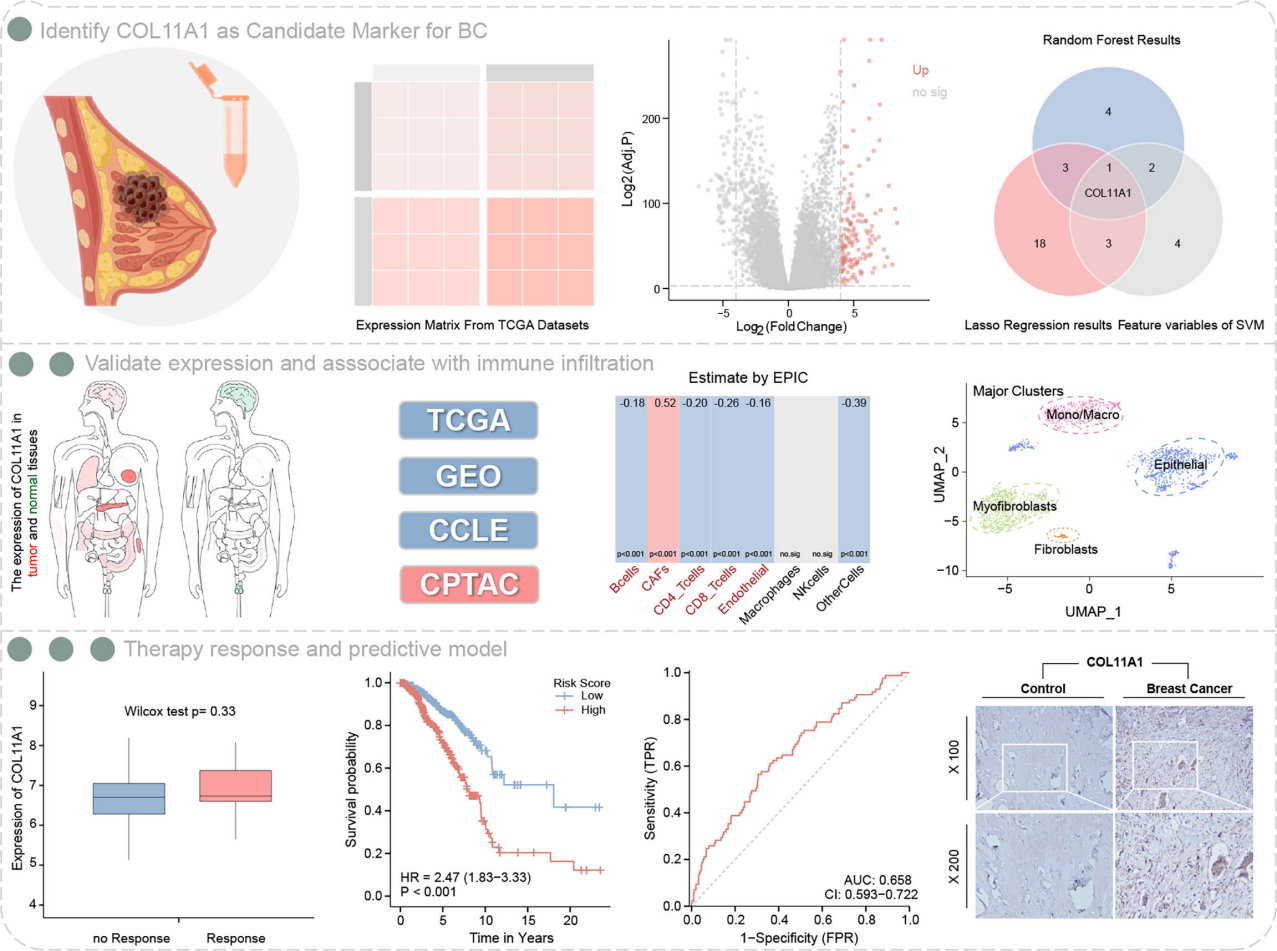
Workflow of this study.

Workflow of this study are shown in **Figure 1**.

### Identification of the hub gene COL11A1

**Figure 2:**
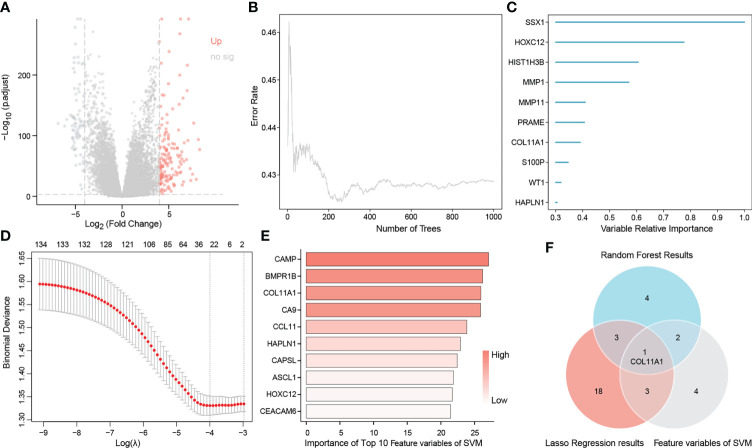
Identification of COL11A1 as a core gene. Volcano showing up-regulated genes **(A)**, random survival forest identified genes of importance associated with patient prognosis **(B, C)**, lasso regression analysis screened feature genes with patient prognosis **(D)**, and support vector machine screened the top 10 feature support vectors associated with prognosis **(E)**, Wayne diagram showing the core genes common to the three machine learning methods COL11A1 **(F)**.

A total of 149 up-regulated genes were identified by the differential analysis of tumor and normal tissues in the TCGA dataset (**Supplementary Table S1**). The results are shown in the volcano map (**Figure 2A**). Random survival forest analysis of these differential genes revealed 11 genes with an importance greater than 0.3 (**Figures 2B, C**). Lasso regression, a machine learning algorithm, was also used for feature variable screening, and a total of 25 candidate genes were selected when the minimum value of lambda was equal to 0.018 (**Figure 2D**) (**Supplementary Table S2**). Conversely, the top 10 feature support vectors obtained by the SVM algorithm, were also selected as candidate genes (**Figure 2E**). Inserting the above results in a Venn diagram, we found that only COL11A1 was common to the results of the three algorithms, and thus this gene was identified as the cores gene of the study (**Figure 2F**).

### COL11A1 is highly expressed in breast cancer samples and is associated with a poor prognosis

**Figure 3:**
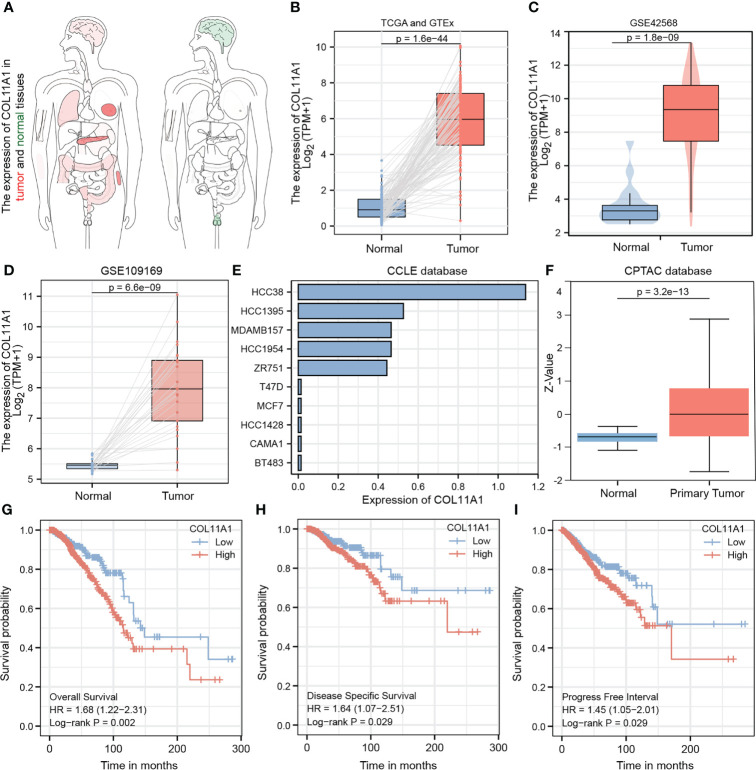
Validated expression of hub gene and Analysis prognosis value. Body map **(A)**, TCGA and GTEx **(B)**, GSE42568 **(C)** and GSE109169 **(D)** confirmed that COL11A1 high expression in tumor tissues.COL11A1 could be highly expressed in HCC38, HCC1395, MDAMB157, HCC1954, and ZR751 cell lines and low expressed in T47D, MCF7, HCC1428, CAMA1, and BT483 cell lines **(E)**. COL11A1 was a high expression in tumor samples **(F)**. COL11A1 was associated with poor prognosis of patients, either Overall Survival, Disease-Specific Survival, or Progress Free Interval **(G–I)**.

The heatmap shows the expression of the COL11A1 gene in normal breast cancer tissues and in different cancer tissues. We found that this gene was significantly highly expressed in breast cancer tissues, while it was almost absence in normal tissues (**Figure 3A**). To further verify this result, we performed an expression difference analysis using tumor samples from TCGA and normal samples derived from GTEx, and obtained consistent results (**Figure 3B**). Furthermore, both GEO datasets, GSE42568 and GSE 109169, also confirmed that the expression of COL11A1 was higher in the tumor compared to normal tissues (**Figures 3C, D**). Furthermore, we also verified that the expression of this gene at the cell line and protein level, and the results suggested that COL11A1 was highly expressed in HCC38, HCC1395, MDAMB157, HCC1954, and ZR751 cell lines and had lower expression in T47D, MCF7, HCC1428, CAMA1, and BT483 cell lines (**Figure 3E**). The results of the protein expression analysis suggested that COL11A1 had higher expression in tumor samples compared to normal tissues (**Figure 3F**). Finally, we performed a survival analysis of this gene and found that high expression of COL11A1 was associated with a poor prognosis in patients, either in terms of OS, disease-specific survival, or progress-free interval (**Figures 3G–I**).

### High expression COL10A1 promoted tumor immune infiltration

**Figure 4:**
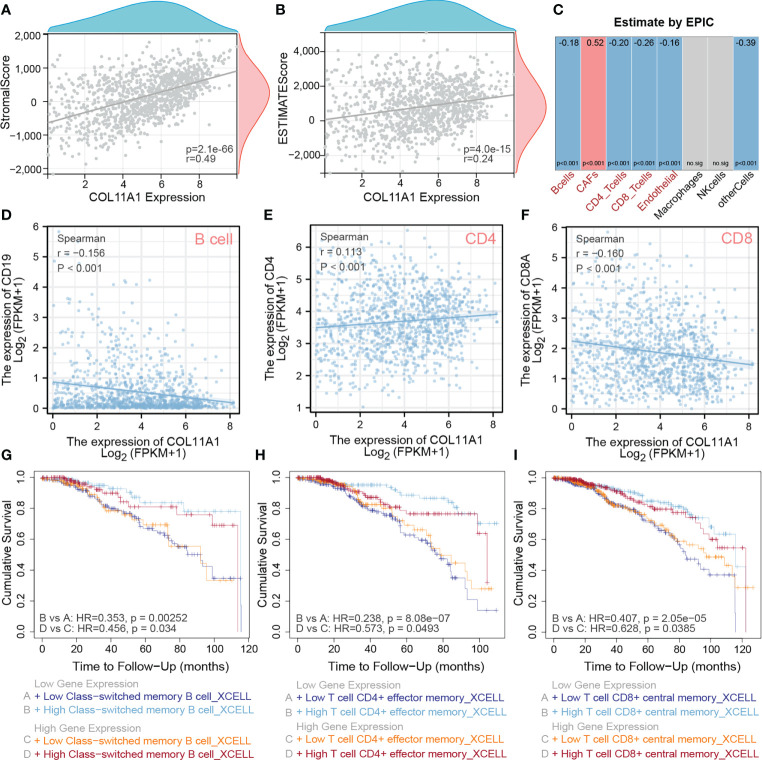
High expression COL11A1 Promote tumor immune infiltration. COL11A1 was significantly positively correlated with a stromal score(r=0.49,p<0.001) and estimate score(r=0.29,p<0.001) in the tumor microenvironment **(A, B)**. COL11A1 expression was negatively correlated with B cells, CD4, and CD8 and positively correlated with CAFs **(C)**. COL11A1 was significantly negatively correlated with the marker of B cells, positively correlated with a marker of CD4, and negatively correlated with a marker of CD8 (r=-0.156, r=0.113 and r=-0.160, respectively; p<0.001) **(D–F)**. The infiltration level of B-cell, CD4 and CD8 cells, were negatively with patient prognosis,no matter COL11A1 expression status **(G–I)**.

The TME is closely associated with tumor progression and metastasis; thus we explored the correlation between this gene and the TME of breast cancer in the current study. The results suggested that COL11A1 expression was significantly positively correlated with the stromal score (r=0.49, p<0.001) and the ESTIMATE score (r=0.29, p<0.001) in the TME (**Figures 4A, B**). Furthermore, the results of immune cell infiltration analysis also revealed that the expression of COL11A1 was negatively correlated with the level of B cells, CD4 and CD8 T cells and positively correlated with CAFs (**Figure 4C**). Further analysis of the correlation between this gene and marker genes of B cells, CD4, and CD8 T cells, revealed that COL11A1 was significantly negatively correlated with a marker of B cells, positively correlated with a marker of CD4 T cells, and negatively correlated with a marker of T cells (r=-0.156, r=0.113 and r=-0.160, respectively; p<0.001) (**Figures 4D–F**). The results of the immune infiltration and survival analysis suggested that in patients with low expression of COL11A1, the degree of B cell infiltration was negatively correlated with patient prognosis. This finding was also applied to the high expression group of COL11A1 expression (**Figure 4G**). However, the level of CD4 and CD8 T cell infiltration was negatively correlated with patient prognosis. These findings further support the association of COL11A1 with tumor immune infiltration and patient prognosis (**Figures 4H, I**).

### High expression COL11A1 positively correlated with CAFs

**Figure 5:**
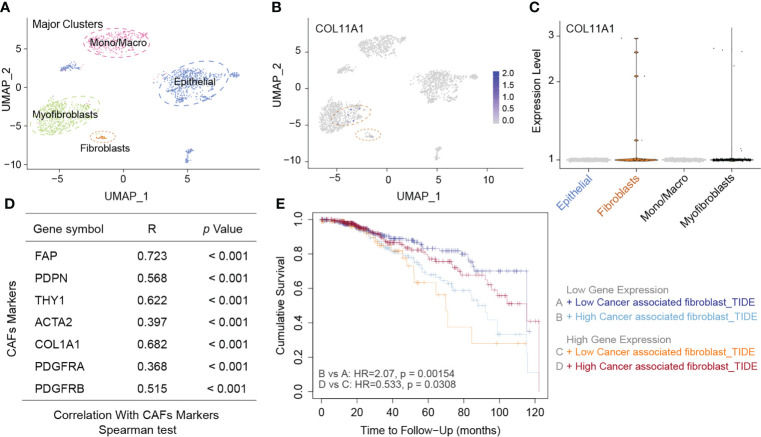
High expression COL11A1 positive with CAFs. Analyzed the distribution of COL11A1 in different cell clusters at the single-cell level, and the data were clustered into four clusters, namely Myofibroblasts, Mano/Macro, Epithelial, and Fibroblasts **(A)**. COL11A1 was most significantly distributed in Myofibroblasts and Fibroblasts. In addition, the expression of this gene was significantly higher in Fibroblasts than in Epithelial cells **(B, C)**. COL11A1 was significantly positively correlated with the CAFs marker gene (FAP, PDPN, THY1, ACTA2, COL1A1, PDGFRA, PDGFRB; p<0.001) **(D)**. The survival analysis results suggested that the deeper the immune infiltration, the worse the prognosis of patients with low expression of COL11A1, and the opposite results of prognosis analysis of patients with COL11A1 high expression **(E)**.

CAFs are present in the tumor stroma and contribute to tumor invasion by promoting the epithelial-mesenchymal transition and participating in tumor angiogenesis. We first analyzed the distribution of COL11A1 in different clusters of cells at the single-cell level, and the results suggested that the data were clustered into four clusters, namely myofibroblasts, Mono/macro, epithelial, and fibroblasts (**Figure 5A**). COL11A1 was distributed most significantly in myofibroblasts and fibroblasts. Additionally, the expression of this gene was significantly higher in fibroblasts than in epithelial cells (**Figures 5B, C**). Further analysis revealed the relationship between COL11A1 and the classical CAF marker gene, and we found that the gene was significantly positively correlated with the CAF marker gene (FAP, PDPN, THY1, ACTA2, COL1A1, PDGFRA, and PDGFRB; p<0.001) (**Figure 5D**). The results of the survival analysis suggested that the deeper the immune infiltration, the worse the prognosis of patients with low expression of COL11A1 and the opposite results of the prognosis analysis of patients with high expression of COL11A1 (**Figure 5E**).

### COL11A1 predicted the response rate to immunotherapy

**Figure 6:**
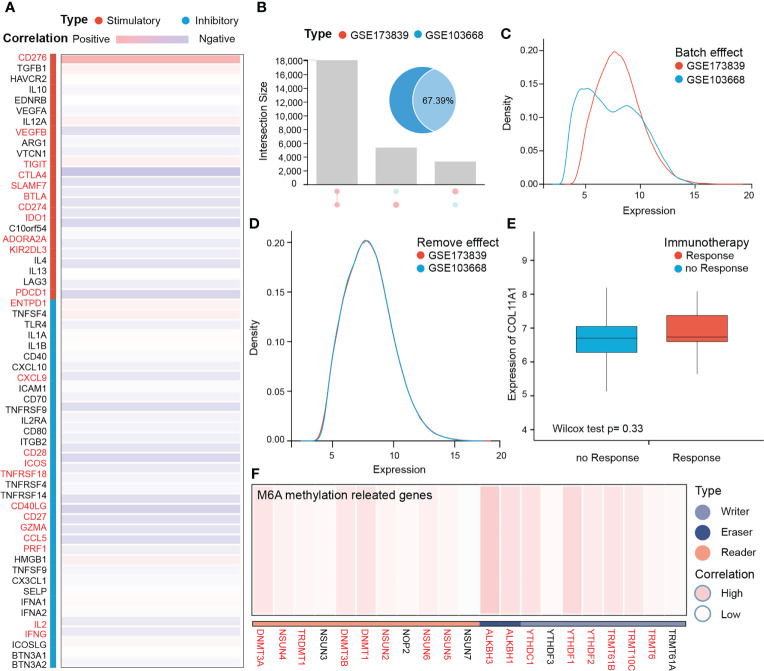
COL11A1 predicts the response rate to immunotherapy. COL11A1 is positively correlated with immune checkpoints (CD276, TIGIT, and ENTPD1;p<0.001) **(A)** Before analysis of two immune therapy datasets have 67.39% genes are overlap, and also have the batch effect, after removing effect, new data with no batch effect **(B–D)**. COL11A1 expression high in the response group, although not significant, when compared with no response samples(p=0.33) **(E)**. In addition, COL11A1 is associated with several m6A methylation-related genes **(F)**.

The results of COL11A1 and immune checkpoints suggest that COL11A1 expression was positively correlated with immune checkpoints (CD276, TIGIT, and ENTPD1; p<0.001) (**Figure 6A**). Further analysis of the results of two immunotherapy data sets revealed that before the analysis of two data sets, 67.39% of the genes overlapped and two data sets had a batch effect, after removal of the effect, new data did not show any batch effect (**Figures 6B–D**). When we analyzed the differences between COL11A1 expression in the response groups and the absence of response groups, we found that COL11A1 showed high expression in the response group, although this was not significant, while compared to the absence of response samples (p=0.33) (**Figure 6E**).

### COL11A1 with m6A methylation

m6A methylation, as a modification of RNA molecules, has become a hot research topic in the life sciences field in recent years. Studies have shown that genes related to m6A methylation promote tumor progression and may mediate tumor immune tolerance. Therefore, we further analyzed the correlation between this gene and m6A methylation-related genes. The results suggest that this gene is associated with several m6A methylation-related genes (**Figure 6F**).

### COL11A1 related immune regulation genes and the construction of a five-gene signature

**Figure 7:**
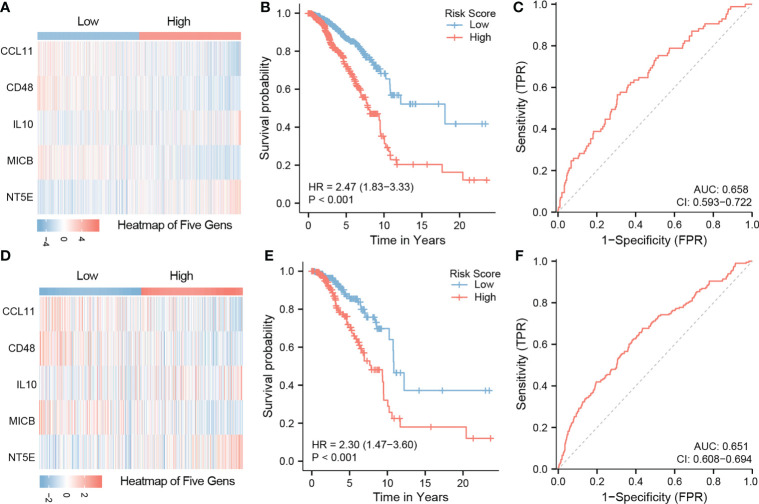
Construction of five gene signature and validation. Constructed a 5 gene signature prognostic model based on the multivarite results. The high-risk group have a worse prognosis than those in the low-risk group (HR=2.47,95%CI 1.83-3.33,p<0.001). The area under the ROC curve of the model was 0.658 **(A–C)**. The internal validation results of the model also demonstrate that high-risk patients the poor outcome while low-risk patients a better ones(HR=2.30,95%CI 1.47-3.60,p<0.001). The area under the ROC is 0.651 in the validation group **(D–F)**.

A total of 43 related immune regulation genes of COL11A1 were identified from breast cancer samples. To determine the prognostic value of these genes, we constructed predictive models using univariate and multivariate Cox regression. The results of the univariate analysis suggested that a total of 19 immune regulation genes were associated with the prognosis (**Supplementary Table S3**), and the multivariate results demonstrate that 5 immune regulation genes were independent risk factors associated with patient outcomes (**Table 1**). Finally, we constructed a 5 gene signature prognostic model based on the above results. We divided patients into the high-risk and low-risk groups based on the median value of the model scores. We found that patients in the high-risk group had a worse prognosis than those in the low-risk group (HR=2.47, 95% CI 1.83-3.33, p<0.001). The area under the model’s ROC curve was 0.658, suggesting that the model had a high predictive value (**Figures 7A–C**). The internal validation of the model also demonstrated that high-risk patients had a poorer outcome than low-risk patients (HR=2.30, 95% CI 1.47-3.60, p<0.001). The area under the ROC was 0.651 in the validation group, indicating that the model was robust (**Figures 7D–F**).

**Table 1:** Multivariate Cox regression for immune regulation genes.

Gene Symbol	Coef	HR	p value	95%CI	
				Lower	Upper
CCL11	-0.137593901	0.871452516	0.040511423	0.76395892	0.994071104
CD48	-0.262096865	0.769436488	0.000509021	0.663727683	0.891981041
IL10	0.453683697	1.574100025	0.000838046	1.206167673	2.054267367
MICB	-0.180198989	0.835104019	0.013780716	0.723540481	0.963869667
NT5E	0.241983075	1.273772634	0.001594269	1.096090998	1.480257321

### Nomogram modeling and efficacy evaluation

**Figure 8:**
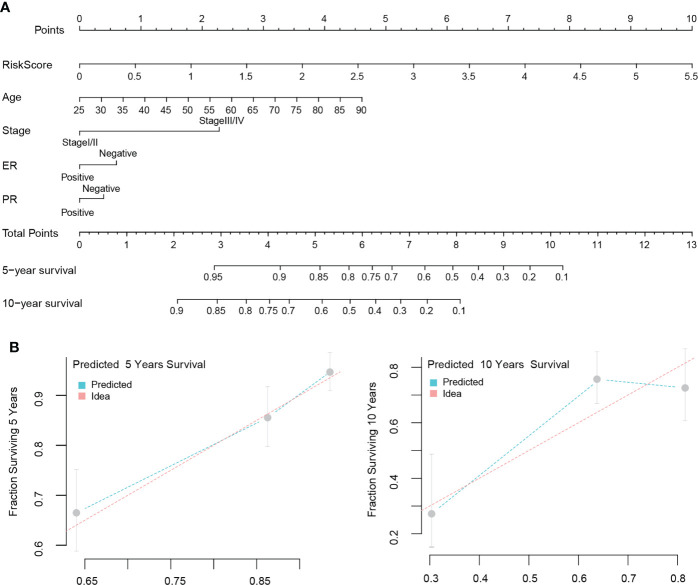
Nomogram modeling and efficacy evaluation. Combine multivariate analysis results with clinical value to construct a nomogram model to predict the OS of patients **(A)**. The 5 and 10 years prediction curve of model had a small bias from the ideal curve **(B)**.

We first evaluated the clinical value of the signature-based on univariate and multivariate COX regression analysis. Univariate results suggested that age, stage, estrogen receptor (ER) positivity, progesterone (PR) positivity, and the risk score could be risk factors that influences the prognosis of patients. The results of the multivariate analysis showed that age, stage, ER status, and risk score were independent prognostic factors for patients (**Table 2**). Finally, we visualized the analysis results to construct a nomogram model to predict the OS of the patients (**Figure 8A**). It is noteworthy that when we built the final version of model, PR status was included, although it showed no significance in the multivariate analysis results. Nonetheless, this variable is very important in clinical decision-making. The C-index of this model is 0.776, and the model correction prediction curve had a small bias from the ideal curve, suggesting that the model could predict the OS of patients at 5 and 10 years with more accuracy (**Figure 8B**).

**Table 2:** Cox Regression analysis for Clinical variables and Signature.

Cox Regression	Univariate Cox regression				Multivariate Cox regression			
Gene Symbol	HR	p value	95%CI		HR	p value	95%CI	
			Lower	Upper			Lower	Upper
Risk Score	2.602	6.56E-09	1.884	3.594	2.591	7.70E-08	1.831	3.668
Age	1.034	1.11E-06	1.020	1.048	1.037	1.76E-07	1.023	1.051
Stage (III/IV vs. I/II)	2.778	9.33E-09	1.960	3.938	3.199	1.07E-10	2.247	4.553
ER (Positive vs. Negative)	0.664	0.0347	0.454	0.971	0.631	0.0196	0.429	0.929
PR (Positive vs. Negative)	0.684	0.0346	0.481	0.973	–	–	–	–

### COL11A1 expression high in breast cancer with clinical samples

**Figure 9:**
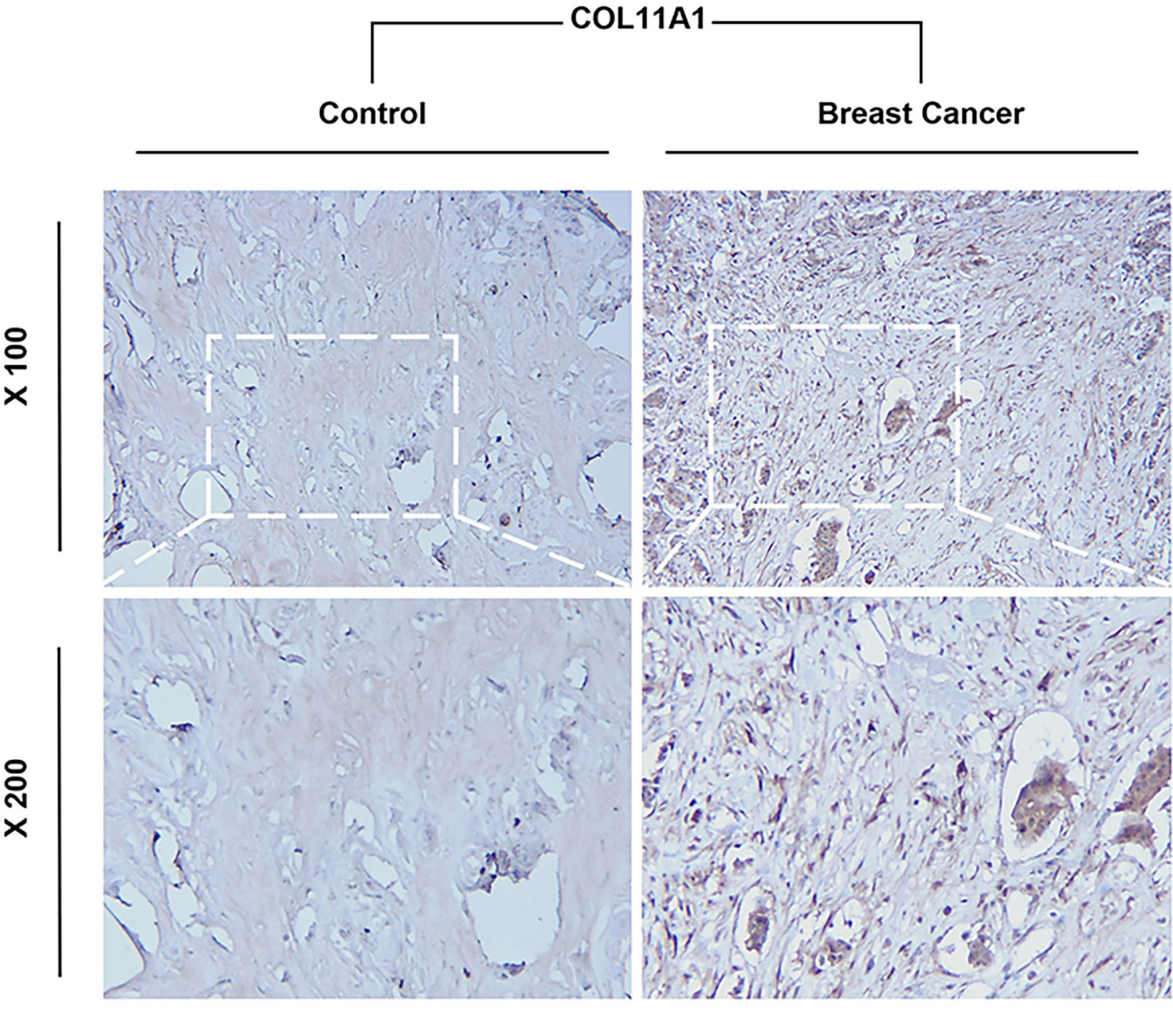
Immunohistochemistry (IHC) results of COL11A1 between normal and tumor breast tissues.

Our Immunohistochemistry (IHC) results demonstrate that COL11A1 could express high in breast cancer tissues while compared with normal tissues in clinical samples (**Figure 9**).

## Discussion

Breast cancer, as one of the three most common tumors in the world, and seriously threatens women’s health worldwide. Although early-stage breast cancer can be successfully treated with surgery, chemotherapy, or combined therapy, more than 30% of patients diagnosed in early-stage will eventually progress and develop an advanced stage (28). Advanced breast cancer is incurable with traditional treatments and has a long-term survival rate of less than 5% (29). These data reveal the urgent need for innovative treatments to reduce relapse and metastasis of breast cancer. The successful application of immunotherapy in a variety of solid tumors and the results of immunological checkpoint antagonists targeting programmed cell death 1 (PD-1) and programmed death ligand-1 (PD-L1) in metastatic breast cancer have raised interest in the area of immune-based strategies for breast cancer (11, 30). Therefore, it is of great significance to explore new immune-related biomarkers to predict treatment response and as a predictor of prognosis (31). We found that COL11A1 was highly expressed at both the transcriptome and protein levels in breast cancer tissues and could serve as a marker of a poor prognosis. Furthermore, we also found that COL11A1 was positively correlated with risk factors in the breast cancer TME. Finally, based on the above results, we identified a COL11A1-associated immunological signature as a predictor in breast cancer.

COL11A1 is located on chromosome 1p21.1, which encodes one of the three alpha chains of type XI collagen and plays a role in the development of skeletal development and fibrillogenesis. But the expression and biological function of COL11A1 in cancers are still controversial and tumor-specific. Some studies have reported that COL11A1 is highly expressed and correlated with poor prognosis in breast cancers, while its expression is low and serves as a good prognostic indicator in some hematological tumors (19). Therefore, more precise mechanisms of COL11A1 should be explored in breast cancers. The composition of immune cells and stromal cells in the TME has been known to play an important role in metastasis, immune escape, and therapeutic resistance in cancers (32). Pearce et al. (33) showed that the COL11A1-related signature was positively correlated with Treg and TH2 in ovarian cancer specimens, demonstrating a poorer prognosis. In a recent study, the high expression of COL11A1 was positively correlated with CD4+T and CD8+T cells, tumor-associated macrophages (TAM), neutrophils and dendritic cells in colon adenocarcinoma, while the function of these immune cells in colon adenocarcinoma TME has not been identified (34). These results suggest that, as one of the components of ECM, COL11A1 may be affected by variable TME in different cancer contexts. The early stage of mammary tumorigenesis is characterized as a stage of acute inflammation, which could activate innate immune cells, such as neutrophils, dendritic cells (DC), and tumor-specific T cells, to eliminate breast cancer cells. While transformed cells escape elimination and a chronic inflammation-like TME is established, which is mainly composed of suppressive immune cells, CAFs, and endothelial cells, leading to immune evasion of advanced breast cancers (35). As shown in our study, COL11A1 had a negative correlation with immune cells (B cells, CD4+ T cells, CD8+ T cells, natural killer cells, and macrophages) in the TME, but showed a positive correlation with CAFs and endothelial cells, which was consistent with the results indicating that overexpression of COL11A1 was only observed in CAF-enriched areas of different cancers and was associated with poorer prognosis (36, 37). All these results implied that COL11A1 could be involved in the tumor immune evasion process and could act as a poor immune-related biomarker in breast cancers.

As representative of ICIs, the Food and Drug Administration has approved treatment with anti-PD-1 and anti-PD-L1 monoclonal antibodies for metastatic triple negative breast cancer (TNbreast cancer) immunotherapy. Faced with these options for breast cancer, it is critical to select potential breast cancer patients populations that could benefit from ICI treatment (14). In addition to PD-L1 expression and tumor mutational burden (TMB), some studies have proposed that TME characteristics could also be used as an indicator to predict response to ICI treatment (38, 39). Furthermore, a retrospective study identified Meflin as serve as a predictive marker of CAF, which could increase the sensitivity to ICI treatment (40). In our study, patients with higher expression of COL11A1 also showed a better response to ICI treatment, which indicates that COL11A1 has candidate potential to predict response to ICIs treatment, in addition to being an immune-related biomarker for prognosis. However, previous studies have shown the opposite predictive role of COL11A1 in response to PD1 checkpoint immunotherapy, reconfirming the heterogeneity and complexity of TME in cancers (34, 41). Thus, it is of great importance to take advantage of multi-omics methods and computational algorithms to interpret the function of genes at the single cell level in different contexts.

With the development of next-generation sequencing technology and computational intelligence techniques, more and more disease markers are being identified, and drugs developed based on these targets will greatly improve patients’ clinical benefits in the future (27, 42–45). In the present study, we used machine learning to identify a new breast cancer marker and further confirm its potential to become a new target. However, relying on a single gene to predict the patient’s prognosis presents drawbacks because, due to the heterogeneity of the disease, disease development can be associated with the abnormal expression of multiple genes. Therefore, we screened five genes related to the immune pathway associated with COL11A1 in breast cancer and constructed a signature to assess the prognosis of the patient based on these genes. This signature also independently predicted patient prognosis compared with patient clinical variables, implying that our multigene signature had high predictive efficacy. In addition, we constructed the NOMOGRAM model, a visual predictive tool based on signature and clinical variables, which, compared with single-gene models and models containing only clinical information. This tool, compared with single-gene models and models containing only clinical information, showed richer predictive properties and greatly enhanced the clinical value of the model.

## Conclusions

We identified COL11A1 as a potential therapeutic target in breast cancer through machine learning, and the high expression of this gene was generally associated with a poor prognosis. Additionally, this gene was also closely associated with breast cancer tumor immune infiltrating cells and could be involved in the tumor immune infiltration process. However, there are some limitations in our study. First, additional machine learning algorithms need to be attempted and elaborately combined to obtain accurate training results. Second, single-cell sequencing data from breast cancer should be included to further clarify the relationship between COL11A1 and the TME in breast cancer. Third, additional clinical RCTs are needed to confirm the predictivity of COL11A1 in the immunotherapy response of breast cancers. Fourth, the possible role of COL11A1 involved in the TME of breast cancers should be further explored through basic research studies.

## Data availability statement

The datasets presented in this study can be found in online repositories. The names of the repository/repositories and accession number(s) can be found in the article/**Supplementary Material**.

## Ethics statement

The studies involving human participants were reviewed and approved by Ethics Committee of Hainan Medical University. The patients/participants provided their written informed consent to participate in this study.

## Author contributions

WS, ZC (2^nd^ author), HL, WP, and CL: Conceptualization, data curation, formal analysis, roles/writing—original draft, writing—review and editing. GW,RF, ZC (8^th^ author), and GC: Roles/writing—original draft. PF, WP and CL: Funding acquisition, methodology, project administration, resources, supervision. All authors contributed to the article and approved the submitted version.

## Acknowledgments

We thank CM for providing experiment validation of COL11A1 with IHC technique.

## Conflict of interest

The authors declare that the research was conducted in the absence of any commercial or financial relationships that could be construed as a potential conflict of interest.

## Publisher’s note

All claims expressed in this article are solely those of the authors and do not necessarily represent those of their affiliated organizations, or those of the publisher, the editors and the reviewers. Any product that may be evaluated in this article, or claim that may be made by its manufacturer, is not guaranteed or endorsed by the publisher.

## Supplementary material

Supplementary Table 1 Different expression genes between tumor and normal with |LogFC| > 4, and p adjust less than 0.01.

Supplementary Table 2 The results of Lasso regression.

Supplementary Table 3 Univariate Cox regression for immune regulation genes.
